# Transcriptome of *Aphanomyces euteiches*: New Oomycete Putative Pathogenicity Factors and Metabolic Pathways

**DOI:** 10.1371/journal.pone.0001723

**Published:** 2008-03-05

**Authors:** Elodie Gaulin, Mohammed-Amine Madoui, Arnaud Bottin, Christophe Jacquet, Catherine Mathé, Arnaud Couloux, Patrick Wincker, Bernard Dumas

**Affiliations:** 1 UMR 5546 Centre National de la Recherche Scientifique (CNRS), Université Paul Sabatier Toulouse III, Université de Toulouse, Pôle de Biotechnologie Végétale, Castanet-Tolosan, France; 2 Genoscope (CEA), Evry, France; 3 UMR 8030 Centre National de la Recherche Scientifique (CNRS), Evry, France; 4 Université d'Evry, Evry, France; Pasteur Institute, France

## Abstract

*Aphanomyces euteiches* is an oomycete pathogen that causes seedling blight and root rot of legumes, such as alfalfa and pea. The genus *Aphanomyces* is phylogenically distinct from well-studied oomycetes such as *Phytophthora* sp., and contains species pathogenic on plants and aquatic animals. To provide the first foray into gene diversity of *A. euteiches*, two cDNA libraries were constructed using mRNA extracted from mycelium grown in an artificial liquid medium or in contact to plant roots. A unigene set of 7,977 sequences was obtained from 18,864 high-quality expressed sequenced tags (ESTs) and characterized for potential functions. Comparisons with oomycete proteomes revealed major differences between the gene content of *A. euteiches* and those of *Phytophthora* species, leading to the identification of biosynthetic pathways absent in *Phytophthora*, of new putative pathogenicity genes and of expansion of gene families encoding extracellular proteins, notably different classes of proteases. Among the genes specific of *A. euteiches* are members of a new family of extracellular proteins putatively involved in adhesion, containing up to four protein domains similar to fungal cellulose binding domains. Comparison of *A. euteiches* sequences with proteomes of fully sequenced eukaryotic pathogens, including fungi, apicomplexa and trypanosomatids, allowed the identification of *A. euteiches* genes with close orthologs in these microorganisms but absent in other oomycetes sequenced so far, notably transporters and non-ribosomal peptide synthetases, and suggests the presence of a defense mechanism against oxidative stress which was initially characterized in the pathogenic trypanosomatids.

## Introduction

The legume root rot disease caused by the oomycete pathogen *Aphanomyces euteiches* is one major yield reducing factor in legume production including pea and alfalfa throughout the world [1]–[3]. *A. euteiches* can infect plants at any age. The first symptoms on pea may be discerned on roots which are softened and water-soaked. The pathogen spreads rapidly through the cortical tissue and the fine branches of feeding rootlets are destroyed. Then the epicotyls become dark and eventually collapse. Leaves progressively turn yellow, starting at the bottom of the shoot. In severe cases, the plants collapse and die before forming any pod.

Thick-walled oospores are resting stages and constitute the primary inoculum source in soil [4]. After germination, primary zoospores are released at the apex of sporangia. These bi-flagellate motile elements encyst at the root surface, germinate and infect the host root. After a few days, the pathogen can reproduce sexually in the host and form new oospores. These oospores can remain dormant in soil and organic debris for many years [5]. Effective chemical controls for *Aphanomyces* root rot of legumes are not available. Crop rotation and bioassay methods to assess the inoculum potential in the soil remain the only effective method to avoid disease. Using recurrent selection-based strategies, pea germplasms with partial resistance or tolerance were obtained [6].

Among Oomycetes, the genus *Aphanomyces* belongs to the Saprolegniales and comprises species that are destructive on plants, crustaceans and fishes such as *A. astaci* and *A. invadans.* Virulent genotypes of *A. astaci* were introduced in Europe giving rise to disease outbreaks among its crustacean host population, the freshwater crayfish [7]. This parasite is now considered as one of the 100 world's worst invasive species (Global Invasive Species Database, http//www.issg.org/database). For the last 30 years, the epizootic ulcerative syndrome (EUS) a disease caused by *A. invadans*, has affected wild and farmed fish in Asia and Indo-Pacific regions and has been recently detected in the eastern seaboard of the US [8]. *Aphanomyces* genus also includes significant plant pathogens such as *A. euteiches* and *A. cochlioides* causing the most serious seedling disease of sugar beet in terms of yield loss, persistence of the oospores in soil, and difficulty of control [9], [10]. By contrast to this diversity of hosts, some oomycete genera, such as the Peronosporale *Phytophthora* is composed of species which are only pathogenic to plants. Such diversity within the oomycetes could reflect different evolutionary histories and possibly different mechanisms of infection between Saprolegniales and Peronosporales [11].

Until recently, oomycetes genomics focussed only on *Phytophthora* spp. with the completion of genome sequencing projects and large scale identification of expressed sequences [12]–[17]. These data, combined to functional studies, led to the identification of new factors of pathogenicity such as the large family of RxLR effectors which are thought to manipulate the plant cell upon their translocation into the host cytoplasm [18]. However little is known about the biology and pathology of other oomycetes, notably those belonging to the Saprolegniales. To fill this gap, we have undertaken a large scale sequencing of *A. euteiches* cDNAs. This microorganism is of interest since it is able to interact with the model legume *Medicago truncatula,* offering the opportunity to engage genetic approaches for deciphering this interaction [9]. Here we report on the characterization of an EST collection comprising 18,684 EST assembled into 7,977 unique sequences from two different cDNA libraries. A systematic comparative strategy was engaged to identify specific *A. euteiches* gene sets absent in *Phytophthora* species. This work greatly expands our knowledge of this group of destructive pathogens and points to the large evolutionary divergence within the oomycete lineage.

## Materials and Methods

### *Aphanomyces euteiches* growth conditions

*Aphanomyces euteiches* (ATCC201684) was maintained on Corn Meal Agar medium (8.5 g/l) at 28°C in the dark. Synthetic medium [19] supplemented with pectin from apple (10 g/l) was used to perform growth assay on pectin.

### cDNA libraries construction

Two unidirectional cDNA libraries from *A. euteiches* were prepared from mycelium grown on medium containing yeast extract/glucose (library MYC) and from mycelium in interaction with *Medicago truncatula* roots (library INT) [20]. A total of 18,684 expressed sequence tags (9,224 from library MYC and 9,460 from library INT) were submitted to the EBI databank for accession number assignation and were assembled into 7,977 unigenes [20].

### Sequence analysis and database searches

Sequences describe here could be downloaded from the *Aphanomyces* database AphanoDB ([19], http://www.polebio.scsv.ups-tlse.fr/aphano/) and cDNA clones are available at the CNRGV (http://cnrgv.toulouse.inra.fr/ENG/). Data searches and analyses have been conducted on AphanoDB using local tools.

For detection of RxLR effectors, all open reading frames (ORFs) encoding at least 100 amino acids (delimited by two stops) were extracted. This led to 136,617 putative ORFs. Signal peptides were predicted with SignalP version 3.0 [21], with a hidden Markov model probability cutoff of 0.8 and 10,131 candidate secreted proteins were obtained.

Three independent strategies were used to look for the RxLR motif over all these sequences:

- Search for the regular expression RxLR-x(1,40)-[ED][ED][KR] (with the fuzzpro program from EMBOSS suite [22]) in the first 100 residues downstream of the signal peptide cleavage site.
This led to the identification of 5 sequences, among which only two were retained as being in the same frame as the BLASTX matches.
- Search with HMM profiles performed with the hmmsearch program from the HMMer 2.2 package [23] with two alternative profiles, RdEER [24] and cropped [18]. From the 2 sequences obtained with this method none was in the frame in agreement with BLASTX matches.
- Search for the regular expression RxLx[EDQ]-x(1,40)-[ED][ED][KR] (with the fuzzpro program from EMBOSS suite) in the first 100 residues downstream of the signal peptide cleavage site. The RxLx[EDQ] motif was the original signal required for secretion identified in *Plasmodium falciparum* [25]: 2 out of the 3 resulting sequences were discarded because of inconsistency of the strand relatively to similarity searches.

Multiple alignments were conducted using the program CLUSTALW [26] and visualized with BOXSHADE (http://www.ch.embnet.org/software/BOX_form.html). CLUSTALW results were submitted to the WebLogo server [27] (http://weblogo.berkeley.edu).

## Results

### cDNA libraries, cDNA sequencing and functional annotation

Two unidirectionnal cDNA libraries were constructed using mRNAs isolated from mycelium of *A. euteiches* cultivated either *in vitro* in a liquid medium (MYC library) or grown in contact to *M. truncatula* roots (INT library). A total of 18,684 high-quality sequences (9,224 for the MYC library and 9,460 for the INT library) corresponding to the 5′ end of cDNA inserts were acquired. A 7,977 unigene set was assembled from EST data. Among the unigenes, 2,843 consensus sequences were assembled from multiple sequence reads and 5,134 were singlets.

**Figure 1:**
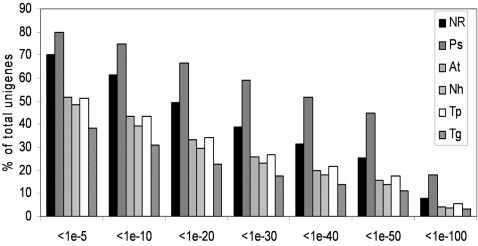
Comparison of *A. euteiches* sequences to the non redundant protein database at NCBI and proteomes of fully sequenced organisms. Unigene sequences were blasted using the BLASTX software to the nr database and to the proteome of *P. sojae* (Ps), *A. thaliana* (At), *N. haematococca* (Nh), *T. pseudonana* (Tp) and *T. gondii* (Tg). For each class of e-value the percentage of unigenes showing homology is indicated.

The 7,977 unigene set was annotated by comparison to the NCBI non-redundant (nr) protein database (5-17-2007 Version) using BLAST analyses [28]. Proteomes of seven fully sequenced organisms were added to the analysis. These include the proteomes from two oomycetes (*P. sojae* and *P. ramorum*), the diatom *Thalassiosira pseudonana*, the pathogenic fungus *Nectria haematococca*, the model plant *Arabidopsis thaliana* and the apicomplexa parasites *Toxoplasma gondii* and *Plasmodium falciparum*. Overall, about 70% of the sequences showed homology to previously described genes in the NCBI non-redundant protein database using a BLASTX E value cut-off of 10^−5^ (Figure 1). As expected, a large proportion of sequences (80%, E value cut-off of <10^−5^) had significant similarities to *Phytophthora* predicted proteins. Interestingly, about the same fraction (50%, E value cut-off of 10^−5^) of *A. euteiches* sequences showed homology to a plant, diatom, apicomplexa or fungal sequence whereas these organisms are distantly related (Figure 1). The unigene set was then annotated by comparisons with the Pfam database of protein domains [29]. 45% of the sequences showed homology with a protein domain with an E value<10^−5^. All the data were stored in a public database called AphanoDB (www.polebio.scsv.ups-tlse.fr/aphano/) which is described elsewhere [20]. Consensus and singlet sequences were named with a unique identifier with the prefix ‘Ae’ for the species, 3 digits for the number of EST included in the contig, the 2 digits ‘AL’ for the strain and 5 digits as unique number.

### Genes highly represented in the interaction library

Determination of the number of sequences specific of each library provided a good indication that the two growth conditions used for library construction allowed the expression of divergent transcriptomes. More than 40% of the sequences appeared to be specific for one library (Table 1). To identify unigenes with a statistical difference in transcript abundance, we used a method described in [30] based upon the frequencies which genes occur in each library and false discovery rate control for multiple test corrections [31]. Eight genes abundantly represented (*p*-value<B-H cutoff) in the interaction library were identified (Table 2). Interestingly, 4 genes encode putative proteins with significant similarity with transporters: 2 sucrose transporters (Ae_48AL7986 and Ae_29AL7339), a protein with an oligopeptide transporter domain (Ae_21AL7132; InterPro domain IPR004813), and a gene coding a probable mitochondrial substrate carrier (Ae_97AL5378). This latter gene is of interest since it has been shown that a gene encoding a mitochondrial carrier protein is required for pathogenicity of the soil-borne fungus *Fusarium oxysporum* [32].

**Table 1:** Number of expressed sequence tags and unigenes specific to a given library.

*Library*	*Singlets*	*Per contigs* a	*Total*	*Specific ESTs (%)*	*Specific unigenes* b
INT	2,932	1,652	4,584	48.40	3,576
**MYC**	2,202	1,772	3,974	43.08	2,755

a Number of ESTs per contigs made of ESTs from a single library.

b Total number of unigenes (singlets or contigs made of ESTs from a single library) found in a given library;

**Table 2:** Unigenes highly represented in the INT library.

Unigene ID	ESTs number INT/MYC	Best BLASTX match^a^	E value	Species	Pfam domain
Ae_97AL5378	93/4	Solute carrier family	5.10^−41^	*Danio rerio*	Mitochondrial substrate carrier
Ae_48AL7986	45/3	Probable sucrose transport protein	7.10^−12^	*A. thaliana*	No hit
Ae_21AL7132	20/1	Metal-nicotianamine transporter	1.10^−70^	*A. thaliana*	Oligopeptide transporter
Ae_29AL7339	27/2	Probable sucrose transport protein	6.10^−13^	*A. thaliana*	No hit
Ae_19AL5526	18/1	No hit			Phosphatidylinositol kinase
Ae_57AL5693	44/13	Elongation factor	7.10^−179^	*Solanum lycopersicum*	Elongation factor tu
Ae_44AL7441	39/5	No hit			No hit
Ae_47AL5766	46/1	No hit			No hit

a BLASTX was done against the SwissProt database

### Genes encoding potential pathogenicity proteins

**Figure 2:**
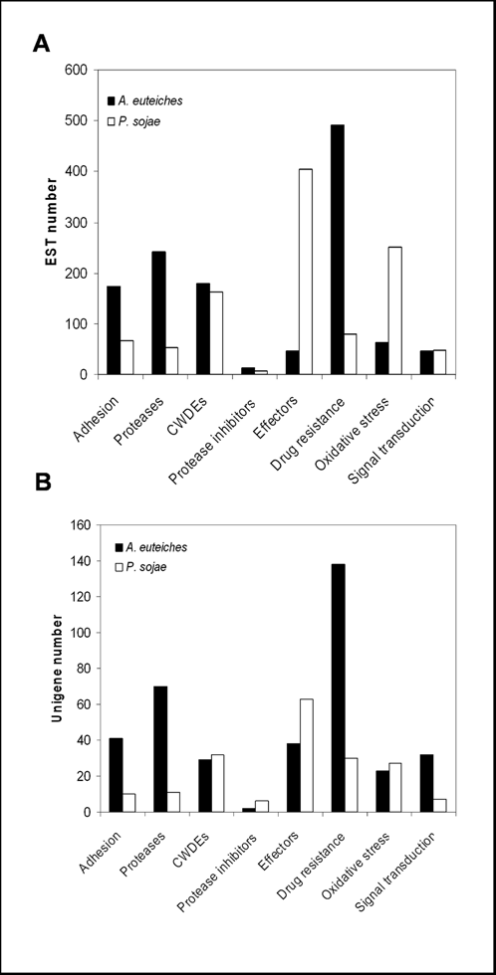
Classification of unigenes based on eight functional categories involved in pathogenicity. *A. euteiches* unigenes (black bars) for which a putative function was assigned were classified into defined categories according to [17] and [16]. For each category the number of ESTs (A) and the number of unigene (B) is shown. For comparison to *Phytophthora*, analysis of a EST collection from *P. sojae* was added to the figure [16] (white bars).

Plant pathogens express a large array of genes playing various roles in pathogenesis. These genes can be roughly classified into eight major categories according to a scheme established for *P. sojae* and *P. parasitica* [16], [17]. To facilitate comparative analyses, *A. euteiches* unigenes were classified following the same criteria. The results obtained with the *A. euteiches* ESTs were compared to those obtained from *P. sojae* ESTs described in [16] since in this later study about the same number of ESTs as in *A. euteiches* was analysed (26,943 ESTs assembled into 7,863 unigenes). This comparative approach revealed striking differences between *A. euteiches* and *P. sojae* (Figure 2). Gene families over-represented in *A. euteiches* compared to *P. sojae* include genes encoding proteins playing a role in adhesion (mucin-like proteins), in protein degradation and in drug resistance (ABC and PDR-like ABC transporter, cytochrome P450 enzymes).

### Adhesion

**Figure 3:**
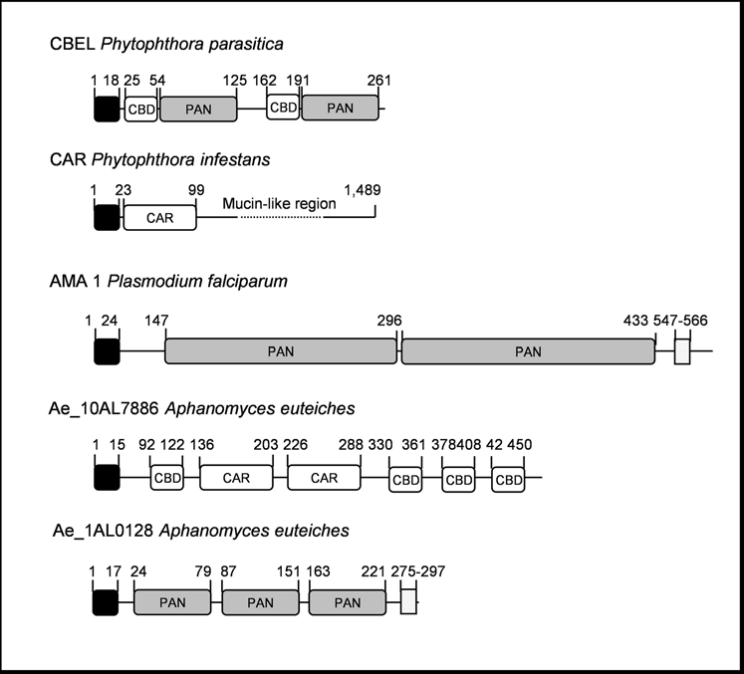
Protein domain organisation of cell surface proteins from oomycetes and apicomplexa. Two proteins from *Phytophthora*, CBEL and CAR which are localized at the cell surface [33], [37] are represented. CBEL contains two closely associated domains, a cellulose binding domain (CBD) and a N/apple PAN domain (PAN). CAR proteins are composed of a mucin-like region and a N-terminal region (CAR) with conserved cysteine residues. AMA1 is a *P. falciparum* protein with two PAN domains [39].

One of the most well-characterized protein playing a key role in oomycete adhesion to plant surface component is a cell wall protein isolated from *P. parasitica* named CBEL (for Cellulose Binding Elicitor Lectin) [33], [34]. CBEL contains two fungal type I Cellulose Binding Domains (CBD or CBM_1; InterPro domain IPR000254) which are involved in cellulose binding but which are also perceived by the host cells as pathogen associated molecular patterns [35]. CBEL also harbours two regions with similarities to the N/apple PAN domain (IPR000177), a conserved domain involved in protein-protein or protein-carbohydrate interactions [36]. cDNA sequences showing typical CBEL features, *i.e* CBDs associated to N/apple PAN domains, were not found in *A. euteiches* ESTs. Accordingly, antigens were not detected with antibodies directed against CBEL, when mycelium extracts were probed by western blot analysis (data not shown). However, CBDs were detected in a large gene family coding proteins showing similarities to a mucin-like protein identified in the soybean root nematode *Heterodera glycines* (GenBank accession number AAC62109). More than 150 ESTs were assembled into 32 unigenes, the largest contig containing 39 ESTs (Ae_39AL5321). Similarity between *A. euteiches* and *H. glycines* proteins is restricted to a domain originally found in cyst germination proteins of *P. infestans* (Figure 3; [37]). The full length sequence of Ae_10AL7886 was determined and domain organization of the predicted protein was deduced (Figure 3). SignalP analysis of the predicted protein identified a 15-amino acids putative signal peptide. Four domains showing typical features of fungal cellulose binding domains were found in the N-terminal and C-terminal ends of the protein. The central part showed similarities to the *H. glycines* mucin-like protein and the cyst germination proteins of *P. infestans*. The combination of these two modules was not detected so far and suggests a role of this protein in cell adhesion.

Eight distinct cDNA sequences showed similarity to CBEL centered on the N/apple PAN domain. This domain is of interest since it has been found in surface proteins of apicomplexan parasites essential for attachment to host cells [38]. Whereas the role of this domain in oomycete proteins is still unclear, it can be speculated that it can interact with host targets to promote pathogenesis. cDNAs coding for proteins with N/Apple domains fell into two categories. The first category included predicted protein sequences for which no other protein domains were found except one or several N/Apple domains. A representative example is Ae_1AL0128 which was fully sequenced (Figure 3). It includes three N/Apple domains and a predicted transmembrane domain located at the C-terminus end. Interestingly, a transmembrane domain was also detected in the *P. falciparum* surface protein AMA1 which was shown to contain two N/Apple PAN domains (Figure 3, [39]). The second category included predicted protein sequences for which N/apple domains were associated with a catalytic domain. Two of these unigenes (Ae_7AL7989 and Ae_1AL5080) were predicted to encode proteins with a peptidase domain associated to one (Ae_1AL5080) or five (Ae_7AL7989) N/apple domains. Finally, three unigenes were identified showing several copies of a thrombospondin type I motif, a conserved 50 amino acid sequence found in surface proteins of *Phytophthora* and apicomplexan parasites [40], [41] and putatively involved in adhesion.

### Cell wall degrading enzymes (CWDEs)

Sequences encoding enzymes potentially involved in degradation of plant cell walls were selected, by looking for the presence of glycoside hydrolase (GH) domains of enzymes hydrolysing substrates that can be found in plant cell walls, such as β-1,4- and β-1,3-glucanases, pectinases, xylosidases, arabinosidases and glucosidases of various specificities [42]. A collection of 53 potential CWDE unigenes out of 7977 unigenes, a proportion similar to what was recorded in *P. sojae* or *ramorum* [15] was obtained. All but one *A. euteiches* CWDE unigenes had a *Phytophthora* homolog with an E value lower than 10^−40^.

β-1,4-glucanases and β-1,3-glucanases mainly of GH families 5 and 81 potentially involved in degradation of plant cellulose and callose were represented by 29 unigenes. Among the putative β-1,3-glucanases from *A. euteiches*, an homolog (Ae_2AL6121) of a previously described GH family 17 endo-1,3-β-glucanase from *Saprolegnia parasitica* [43] and *P. infestans* (PiENDO1; [44]) was found, as well as two unigenes (Ae_2AL7302 and Ae_12AL7443) showing high similarity to the GH family 5 exo-1,3-β-glucanase PiEXO3 from *P. infestans* [44]. Interestingly, the latter unigenes are exclusively composed of ESTs from the interaction library, suggesting that they are involved in the interaction with the plant.

Other glycosidases from *A. euteiches*, potentially involved in degradation of plant cell wall polysaccharides such as xyloglucans, xylans or galacto(gluco)mannans, were represented by 24 unigenes encoding GH family 1, 3 and 30 enzymes. However, GH family 12 endoglucanases, potentially involved in xyloglucan degradation and represented by a family of 8 to 10 genes in *P. ramorum* and *P. sojae* [45], and GH family 10 xylanases, represented by more than 5 genes in the *P. ramorum* and *P. sojae* genomes, were missing in the database.

**Figure 4:**
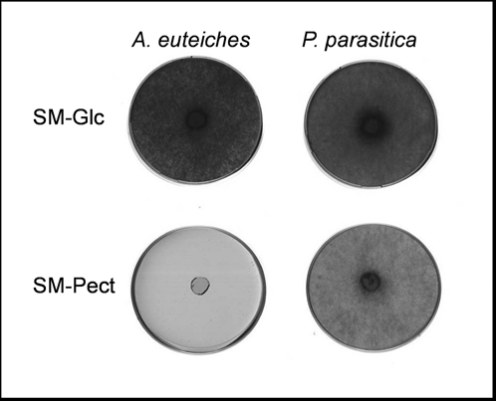
Growth of *A. euteiches* on synthetic media. *A. euteiches* and *Phytophthora parasitica* mycelium were grown on synthetic medium containing Glucose (SM-Glc) or Pectin (SM-Pect) as carbon source. Photos were taken 15 days after inoculation.

Surprisingly, no EST related to pectin metabolism was found, despite the fact that *P. sojae* and *P. ramorum* genomes contain numerous pectinase genes [15], and *bona-fide* pectinase genes were found in *P. infestans*, *P. cinnamomi* and *P. parasitica* [12], [46]–[48]. The lack of sequences related to pectin degradation is correlated with the lack of pectinase activity observed in yeast extract/glucose medium (data not shown) and the failure to grow *A. euteiches* on a synthetic medium containing pectin as sole carbon source, whereas *P. parasitica*, which produces an inducible polygalacturonase [47], grew equally well on glucose or pectin medium (Figure 4). These results showed that pectinase genes were poorly expressed in the growth conditions we used, and that pectinase genes are differently expressed in *A. euteiches* and *Phytophthora* species.

### Proteases and protease inhibitors

A large set of *A. euteiches* sequences were predicted to encode proteases (Figure 2). Serine, aspartyl and cysteine proteases were identified, the largest family being the cysteine protease family. Comparison with the *P. sojae* ESTs collection revealed that the number of protease genes is significantly higher in *A. euteiches* than in *P. sojae*. For example, 17 unigenes (67 ESTs) of *A. euteiches* were predicted to encode serine carboxypeptidase (IPR001563) whereas one unigene (1 EST) was found in *P. sojae*. Another example is represented by cysteine proteases (IPR000668) for which 25 unigenes (125 ESTs) were found whereas only 5 unigenes (41 ESTs) were detected in the *P. sojae* EST collection. Moreover, two types of proteases belonging to the cysteine protease family, peptidase c1b (IPR004134) and peptidase c14 (caspase, IPR011600) were found only in *A. euteiches* and not in other oomycetes. This analysis showed that *A. euteiches* is clearly distinct from *Phytophthora* species with respect to protease production, and this is in accordance with the detection of a high protease activity in culture medium (data not shown).

**Figure 5:**
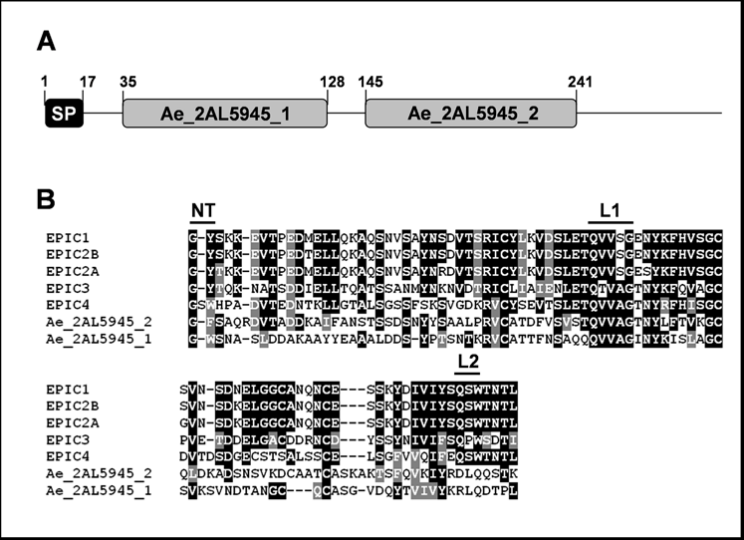
A cystatin-like protease inhibitor in *A. euteiches.* A, domain organisation of the deduced protein sequence of Ae_2AL5945. A putative signal peptide (SP) and two domains similar to cystatin domain are shown. B, alignment of cystatin domains from *P. infestans* cystatins (EPIC1, EPIC2A, EPIC2B , EPIC3 and EPIC4) and from Ae_2AL5945. The active sites of cystatins, including the N-terminal trunk (NT), first binding loop (L1), and second binding loop (L2), are shown.

By contrast with the large number of proteases genes, only two genes (13 ESTs) encoding protease inhibitors were detected. These include a cystein protease inhibitor of the cystatin family (Ae_2AL5945; IPR000010) and a serine protease inhibitor (Ae_11AL6547) containing Kazal domains (IPR002350). These two types of protease inhibitors and their potential role in defense-counterdefense mechanisms were studied in *P. infestans* [49], [50]. Five domains containing typical features of Kazal motifs were found in Ae_11AL6547. However, more domains could be present since the sequence appeared to be incomplete. The predicted translation product of Ae_2AL5945 were analysed with SignalP leading to the identification of a 17 amino acid signal peptide with a significant mean S value of 0.90 and hidden Markov probability of 1.0. Similarity searches of the predicted protein against the NCBI non-redundant database using the BLASTP program revealed a weak similarity (*E* value = 6.10^−3^) to the *P. infestans* cystatin EPIC4, centered on the predicted active site. Multiple alignment using ClustalW [26] was done using cystatin domains from *P. infestans* (EPIC1, EPIC2A, EPIC2B, EPIC3 and EPIC4) and the two domains from Ae_2AL5945 (Ae_2AL5945_1 and Ae_2AL5945_2). Cystatin domains from *A. euteiches* are clearly distinct from *P. infestans* sequences except in the highly conserved sites of cystatins, *i.e.* the N terminal trunk and the L1 binding loop (Figure 5). However, the second binding loop is divergent in *A. euteiches* sequences.

### Effectors

A class of oomycete effectors which could be delivered inside plant cells during infection was recently characterized. Effectors from *Hyaloperonospora parasitica*, *P. infestans* and *P. sojae* do not show extensive similarity except a conserved RxLR motif downstream of the signal peptide, followed by an EER motif. The *A. euteiches* unigene collection was mined for putative RxLR effectors looking for matches either to a sequence pattern or to HMM profiles. We end up with two unigenes containing the RxLR-dEER motif (Ae_1AL5059, Ae_1AL1390) and one with a RxLx[EDQ]-dEER motif (Ae_40AL5333), which is close to the *Plasmodium* motif. Ae_1AL5059 and Ae_1AL1390 showed homology to a putative *P. sojae* and *P. ramorum* genes but appeared to be incomplete. Ae_40AL5333 encodes a putative lysine rich protein with no homology to known protein sequence.

**Figure 6:**
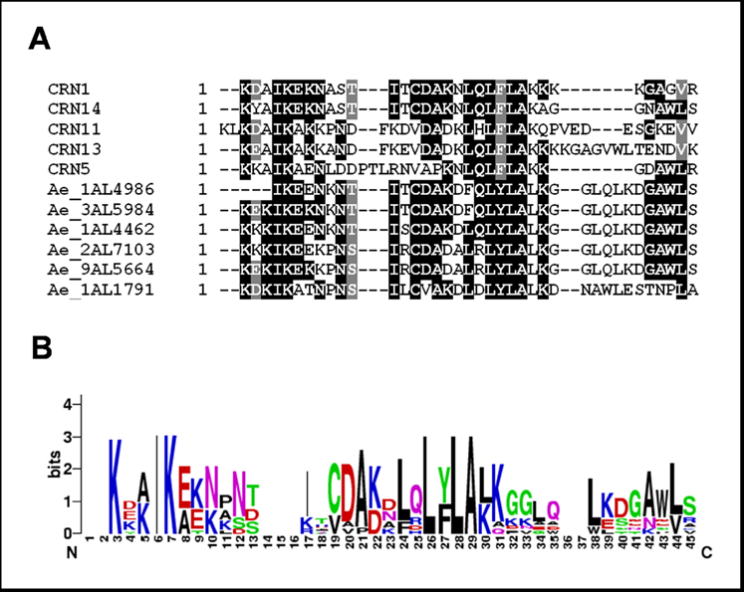
Identification of conserved amino acid residues at the N-terminal extremities of *P. infestans* and *A. euteiches* CRN sequences. A, multiple alignment of *A. euteiches* like CRNs and *P. infestans* CRNs (CRN1, CRN5, CRN11, CRN14) showing the highest homology to *A. euteiches* sequences. B, consensus sequence pattern calculated using Weblogo showing the most conserved amino acid residues.

A distinct class of putative cytoplasmic effectors is the Crinkling and Necrosis (CRN) family first described in *P. infestans* [48]. While *Phytophthora* CRNs lack an RxLR motif, some *H. parasitica* CRNs show an RxRL sequence overlapping another conserved motif LxFLAK [24]. Several sequences showing high similarity with *Phytophthora* CRN were detected in *A. euteiches*. The largest groups of CRNs showed strong similarity to *P. infestans* CRN5 (14 unigenes) or CRN13 (9 unigenes). Interestingly, the conserved LxLFLAK sequence was not present in *A. euteiches* genes, but a closely related motif, F/LxLYLALK, was detected (Figure 6). This is the first time that CRN genes are found in an organism distinct from Peronosporales suggesting that this class of effectors plays an important role in oomycete pathogenicity.

Other oomycete effector genes include genes coding necrosis-inducing peptides (NPP family; [51], [52]) and elicitins [53], [54]. No ortholog of NPP was found and only one divergent sequence encoding a putative elicitin-like protein was detected (Ae_1AL4317).

### Transporters

A large number of sequences were predicted to encode proteins involved in transport of molecules. The largest family encodes ABC transporters (84 unigenes and 342 ESTs) and PDR-like ABC transporters (28 unigenes and 86 ESTs). Interestingly, two oligopeptide transporter genes (Ae_21AL7132 and Ae_1AL0658) encoding putative proteins with an OPT domain (IPR004813) were found. Two distinct, proton-coupled peptide transport systems have been described in eukaryotic organisms: the PTR (peptide transport) system, specific for di- and tripeptides, and the OPT (oligopeptide transport) system, which transports tetra- and pentapeptides. Homolog of di-/tripeptide transporters are found in virtually all organisms, whereas the oligopeptide transporters are limited to fungi and plants (reviewed in [55], [56]). While the PTR system is well represented in *Phytophthora* proteomes (8 genes in *P. sojae*), the OPT proteins are absent, suggesting that OPT dependant oligopeptide transport is not widely found in oomycetes. However, Ae_21AL7132 and Ae_1AL0658 show strong similarity with an *A. thaliana* sequence (E value = 6.10^−72^ and 5.10^−38^ respectively) encoding a metal-nicotianamine transporter. This class of plant transporters is involved in iron–phytosiderophore uptake by roots but exhibits also significant sequence similarity to several functionally characterized yeast transporters of the OPT family [57], [58]. Thus it cannot be excluded that Ae_21AL7132 and Ae_1AL0658 encoded proteins are involved in uptake of phytosiderophore-Fe complex. Interestingly, ESTs assembled into Ae_21AL7132 were found almost exclusively in the interaction library (20 ESTs in the interaction library and 1 in the mycelium library; Table 2) suggesting a role for this class of transporters in pathogenesis.

### Identification of *A. euteiches* genes without orthologs in *Phytophthora*

**Figure 7:**
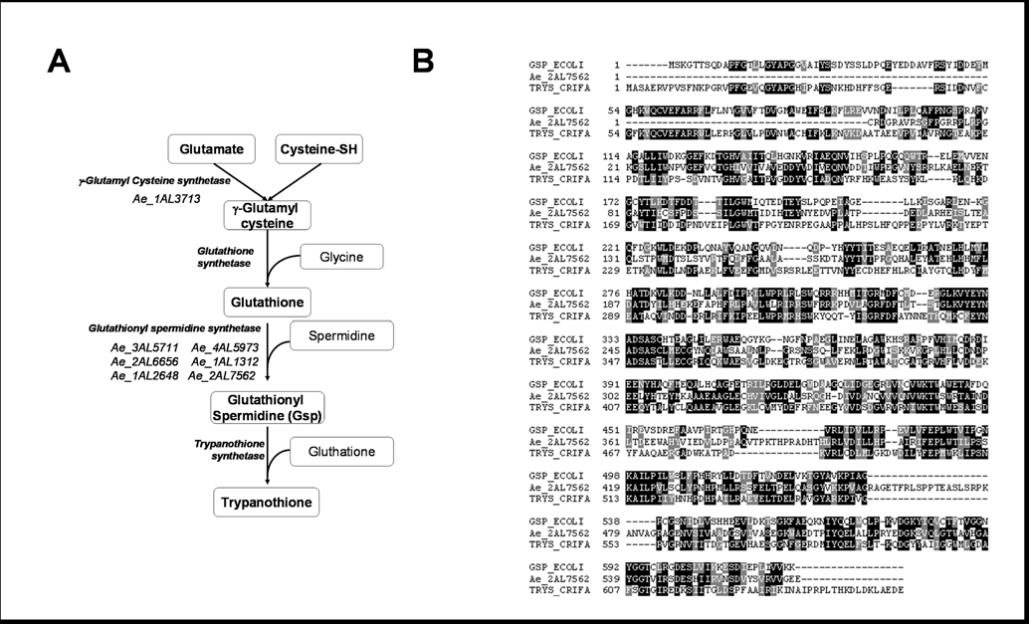
Identification of *A. euteiches* genes putatively involved in trypanothione synthesis. A, trypanothione biosynthesis starts with the formation of glutathione catalysed by g-glutamyl synthetase and glutathione synthetase. Trypanothione is synthesized from glutathione and spermidine either in two successive steps catalysed by glutathionyl spermidine synthase and trypanothione synthase. It has been shown in some cases that trypanothione synthase is able to catalysed the two reactions. Unigenes predicted to code enzymes belonging to this pathway are indicated. B, multiple alignment of gluthationyl spermidine synthetase from *E. coli* (GSP_ECOLI), from *Crythidia fasciculata* (TRYS CRIFA) and Ae_2AL7562.

Identification of unigenes without any similarity to *Phytophthora* sequences but with similarity to sequences from trypanosamatid parasites led to the identification of a family of proteins putatively involved in the synthesis of trypanothione, a compound playing a key role in defense against oxidative stress in trypanosomatids [59]. Trypanothione is synthesized by conjugation of the polyamine spermidine and the tripeptide glutathione. The biosynthesis of trypanothione starts with the formation of glutathionylspermidine (Gsp) which then reacts with a second GSH molecule to form trypanothione (Figure 7A). These two biosynthetic steps are catalysed by two distinct enzymes, glutathionylspermidine synthetases and trypanothione synthetases. However, it has been shown that trypanothione syntethase can catalyse the entire synthesis of trypanothione in *Trypanosoma cruzi* and *Crithidia fasciculata* [60], [61]. Glutathionylspermidine has also been identified in *E. coli* where it could be involved in regulating the intracellular level of spermidine and glutathione. Six *A. euteiches* unigenes were found encoding proteins with a typical glutathionylspermidine syntethase domain (IPR005494) and similarity to trypanothione syntethase sequences from trypanosomatids (E value<10^−10^). Moreover, one sequence (Ae_2AL7562) contains a second domain found in trypanothione synthetases and bacterial bifunctional glutathionylspermidine synthetase/amidase, the CHAP (cysteine, histidine dependant amidohydrolase/peptidase) domain (IPR007921). Alignment of the protein sequence with an *E. coli* bifunctional glutathionylspermidine synthetase [62] and a trypanothione synthetase from the insect parasite *C. fasciculata* [63] is shown on Figure 7B. These results suggest the presence of a glutathionylspermidine based defense mechanism against oxidative stress in *A. euteiches* that is absent in other oomycetes such as *P. sojae* and *P. ramorum*.

To find *A. euteiches* genes which could be absent in other oomycetes but present in fungal plant pathogens, all the unigenes sequences were compared to the pea root pathogen *N. haematococca.* The sequences showing an E value greater than 0.1 to a *Phytophthora* sequence and lower than 10^−10^ to a sequence from the *N. haematococca* proteomes were selected. About half of the sequences (19 unigenes) matched to sequences of unknown function and without known protein domains. Interestingly, 3 of the remaining sequences (Ae_1AL0272, Ae_2AL7741 and Ae_1AL3374) showed a strong similarity to hydrolytic enzymes (glucan hydrolases and lipase) and 3 (Ae_1AL1763, Ae_1AL3400 and Ae_1AL0931) to non-ribosomal peptide synthetases (NRPS). These latter enzymes are of outstanding importance since their products have been shown to be involved in fungal pathogenesis [64], [65] (Table 3). Four putative NRPS genes were detected in *P. sojae* and *P. ramorum* genomes [15]. However these genes are not related to the *A. euteiches* sequences. These data suggests that *A. euteiches* could be able to produce secondary metabolites distinct from those synthesized by *Phytophthora* species.

**Table 3:** *A. euteiches* unigenes showing a BLASTX match to fungal, plant or diatom proteins (E value<1.10^−10^) and not to *Phytophthora* proteins (E value>0.1).

Unigene ID	Best hit	Best BLASTX match nr databank	E value	Species	Pfam domain
Ae_1AL1763	*N. haematococca*	nonribosomal peptide synthetase	2.10^−35^	*Omphalotus olearius*	Condensation domain
Ae_1AL0931		non-ribosomal peptide synthetase	8.10^−46^	*Granulibacter bethesdensis*	No hit
Ae_1AL3400		unnamed protein product	1.10^−17^	*Aspergillus oryzae*	Condensation domain
Ae_1AL0272		β 1,3-glucanase	3.10^−19^	*Strongylocentrotus purpuratus*	No hit
Ae_2AL7741		hypothetical protein	1.10^−36^	*Phaeosphaeria nodorum*	GH 71
Ae_1AL3374		hypothetical protein	1.10^−14^	*Gibberella zeae*	Lipolytic enzyme
Ae_2AL7237	*A. thaliana*	hypothetical protein	9.10^−23^	*Oryza sativa*	HMGCoA reductase
Ae_8AL7813		HMG-CoA reductase	6.10^−98^	*Gossypium hirsutum*	No hit
Ae_1AL1432		HMG-CoA reductase	5.10^−82^	*Gossypium hirsutum*	HMGCoA reductase
Ae_1AL0818		cycloartenol synthase	5.10^−88^	*Betula platyphylla*	No hit
Ae_1AL0450		thiazole biosynthetic protein	1.10^−110^	*Nicotiana tabacum*	thiamine biosynthesis thi4 protein
Ae_1AL1432	*T. pseudonana*	HMG-CoA reductase	5.10^−82^	*Gossypium hirsutum*	HMGCoA reductase
Ae_2AL7237		hypothetical protein	9.10^−23^	*Oryza sativa*	HMGCoA reductase
Ae_3AL7274		lanosterol synthase	8.10^−72^	*Homo sapiens*	Prenyltransferase/squalene oxidase
Ae_2AL7738		unnamed protein product	8.10^−63^	*Tetraodon nigroviridis*	Squalene/phytoene synthase

Comparative analysis of *A. euteiches* sequences with a high BLASTX score to a *A. thaliana* or *T. pseudonana* sequences allowed the identification of two biosynthetic pathways which are absent in *Phytophthora* species but present in *Aphanomyces* (Table 3). One sequence (Ae_1AL0450) showed significant similarity to a thiamine biosynthetic enzyme from plants (top hit to protein AAP03875, E value = 1.10^−110^) and a thiamine biosynthesis Thi4 protein domain (IPR002922). This enzyme is involved in the synthesis of the thiamine precursor, thiazole. Members of the genus *Phytophthora* are thiamine auxotrophs and consequently lack this biosynthetic enzyme whereas *A. euteiches* and other Saprolegniales such as *Saprolegnia parasitica* are able to synthesize thiamine. Accordingly, the sequence from *A. euteiches* is highly similar to the *S. parasitica* gene (SPM2G2; 93% identity at the protein level) described in [43].

*Phytophthora* species being sterol auxotrophs, key genes coding for enzymes involved in sterol biosynthesis were not identified in *Phytophthora* genomes [15]. By contrast, Saprolegniales species such as *A. euteiches* are able to grow in minimal media without exogenous sterols. Accordingly, several genes coding for putative sterol biosynthesis enzymes were identified. These include two cycloartenol synthases (Ae_3AL7274; Ae_1AL0818) and a cytochrome P450 enzyme belonging to the CYP51 family (Ae_5AL7244) which strongly matched (E values<10^−70^) plant enzymes. Each gene has also closed orthologs in the diatom *T. pseudonana* suggesting that the sterol biosynthetic is ancient in the oomycete lineage and has been lost by *Phytophthora* species.

## Discussion

In this study we describe an extensive characterization of an EST collection of *A. euteiches*, an economically important plant pathogen. The closest oomycete specie which was the subject of a genomic approach is the fish pathogen *S. parasitica*, with the sequencing of 1,510 cDNA clones (1,279 unigenes) obtained from one library constructed from mycelium grown axenically [43]. The current study is more expansive in terms of number of cDNA clones sequenced (18,684) and constructed libraries (2) and adds to the EST resources available for other oomycetes such as *P. sojae* (7,863 unigenes) [16], *P. infestans* (18,256 unigenes) [12] and *P. parasitica* (4734 unigenes from two libraries) [17], [66]. Comparison between the unigene contents of the two *A. euteiches* libraries showed that they reflected two divergent expression programs. The procedure used to cultivate *A. euteiches* mycelium in contact to *M. truncatula* roots avoided contamination of pathogen mRNAs with plant mRNAs [20]. Nevertheless, perception of plant root tissue by the pathogen occurred, leading to the induction of a specific subset of genes. These include 4 transporter genes which could be involved in nutrient uptake during infection.

Comparative analysis of *A. euteiches* unigene sequences to proteomes derived from fully sequenced organisms provided a global view of sequence similarities distribution among organisms which are distantly related. As expected, most of sequences showed similarity to a *Phytophthora* predicted protein sequence. About 20% of unigenes did not have any ortholog in *P. sojae* or *P. ramorum*. More surprisingly, we found that similar proportions of unigenes have an ortholog in the plant *A. thaliana*, the fungus *N. haematococca*, the diatom *T. pseudonana* and the apicomplexa *T. brucei*. These sequences were examined in more detail with a particular emphasis to those with no orthologs in *Phytophthora* genomes. This revealed the presence of genes involved in biosynthetic pathways which are known to be absent in *Phytophthora*. For example, it is known that *Phytophthora* species, but not *Aphanomyces* species, are thiamine auxotrophs. Accordingly, a gene coding a thiamine biosynthetic enzyme was identified, showing homology with a *S. parasitica* sequence [43]. Another highly significant example concerns sterol metabolism. Among oomycetes, members of the Saprolegniale order can synthesize sterols *de novo* whereas species belonging to Pythiales are sterol auxotrophs. We found several genes encoding proteins showing high similarity to enzymes involved in sterol biosynthesis. This pathway is a major target for chemical inhibitors and a large number of fungicides have been developed acting mainly on a demethylation step catalysed by a cytochrome p450 enzyme of the CYP51 family. The identification of this class of proteins in *A. euteiches* will allow the development of new compounds acting on Saprolegniale pathogens. The ability of *A. euteiches* to synthesize its own sterols could be correlated with the fact that one elicitin-like sequence has been detected and no antigen was detected with anti elicitin antibodies when culture filtrates were probed by western blot analysis (data not shown). Elicitins are proteins found only in oomycetes where they were proposed to act as sterol carriers [67]. In *Phytophthora*, elicitins represent a major class of secreted protein and are highly expressed. For example, 210 ESTs, corresponding to 16 elicitin genes were detected in the *P. sojae* EST collection [16]. Moreover, elicitins can be perceived by the host cell, leading to a strong induction of defense reactions, and are thought to play a role in plant-pathogen specificity [53]. Thus, the low expression of elicitins in *A. euteiches* could be linked to its ability to synthesize its own sterols.

Classification of putative pathogenicity factors into 8 categories revealed other striking differences between *A. euteiches* and *Phytophthora* species. These include qualitative and quantitative differences for gene encoding hydrolases, effectors or proteins involved in adhesion, drug resistance and oxidative stress. Adhesion of pathogens to the surface of host tissues is a critical step in the establishment of oomycete pathogenesis [68]. Although several adhesins produced by fungal pathogens have been characterized, molecular mechanisms involved in adhesion of oomycetes plant pathogens are still largely unknown. These include cyst germination proteins from *P. infestans* which share homology with mucins [37], *Phytophthora* surface proteins containing thrombospondin repeats [40] and *P. parasitica* CBEL [34]. A functional approach demonstrated the essential role of CBEL in adhesion. Inhibition of CBEL gene expression in transgenic *P. parasitica* strains led to a dramatic reduction in adhesion of the mycelium to cellulosic substrates [69]. Several sequences showing similarity with *Phytophthora* adhesins were found in *A. euteiches* but with specific features. Of particular interest is a highly expressed family of proteins composed of a combination of cellulose binding domains (CBM_1) similar to those present in CBEL, and acidic domains found in the N-terminal part of the *P. infestans* cyst germination proteins. These proteins can represent a novel class of oomycete adhesins.

Analysis of the EST collection revealed expansion of gene families in *A. euteiches* such as genes encoding proteases. Proteases and protease inhibitors play an important role in the molecular dialogue which establishes between the pathogen and its host. In *Phytophthora*, several protease inhibitors have been shown to target plant proteases during infection [49], [50]. Expansion of proteases gene families in *A. euteiches* indicates that these proteins are probably major players of *A. euteiches* pathogenicity. Thus, protein degradation and utilization is probably a major metabolic pathway related to pathogenesis in *A. euteiches*. Others genes showing expansion and diversification in *A. euteiches* comprise ABC transporters whose role in mediating resistance to plant toxins and fungicide has been suggested in *Phytophthora* [70]. By contrast, some gene families which are well represented in *Phytophthora* were not detected in *A. euteiches*. This is the case for pectinases for which their role as pathogenicity factors has been demonstrated for several plant pathogens. The lack of ESTs showing homology to pectinases is correlated to the weak production of extracellular pectinolytic activity *in vitro* and the absence of *A. euteiches* growth on a medium containing pectin as sole carbon source. Recently, sequencing of a 192 bp genomic DNA fragment of *A. euteiches* revealed similarity with a *P. capsici* polygalacturonase [71]. Thus it cannot be definitively concluded from our results that pectinase genes are not present in *A. euteiches*.

Another group of oomycete pathogenicity factors are proteins which are translocated into the cytoplasm of the host cell during infection. These gene products all contain an RxLR motif which has been shown to be essential for the effective transport of these effectors [18]. While no clear homolog of effectors belonging to the RxLR class were identified in *A. euteiches*, their presence cannot be fully excluded. It has been shown that these proteins are under a strong positive selection [24] and can thus escape *in silico* analyses. However, genes showing strong similarity with a second class of effectors belonging to the crinkler family were identified. Up to now, these proteins were found only in *Phytophthora* species where they represent a large superfamily with 40 genes in the genome of *P. sojae* [15]. CRNs were issued from a large screen aiming at identifying extracellular proteins from *P. infestans* able to induce symptoms when expressed in plant tissues [48]. While the exact function of CRNs is still unclear, conservation of these sequences in all pathogenic oomycetes examined so far indicates that they probably play a specific role in oomycete pathogenesis.

Finally, comparison of *A. euteiches* sequences to protein sequences from tryponosomatids led to the identification of protein sequences with strong similarity to enzymes involved in synthesis of antioxidant compounds, glutathionylspermidine and trypanothione. Trypanothione has been identified as an essential redox intermediate and its role in defense against oxidative stress has been demonstrated in the human pathogens *Trypanosoma brucei* and *Leishmania donovani,* [72], [73]. Since the oxidative burst is one of the earliest observable plant defense response against pathogen invasion (for review see [74]), the production of antioxidant compounds will constitute an efficient defense mechanism for the pathogen.

In conclusion this work greatly expands our knowledge of a genus which includes plant and animal pathogens and more generally illustrates the diversity which can exist among the oomycete. The repertoire of expressed genes described here will be used as source of candidate genes for functional studies aiming at identifying essential factors of *A. euteiches* pathogenicity.
